# eTPM: A Trusted Cloud Platform Enclave TPM Scheme Based on Intel SGX Technology

**DOI:** 10.3390/s18113807

**Published:** 2018-11-06

**Authors:** Haonan Sun, Rongyu He, Yong Zhang, Ruiyun Wang, Wai Hung Ip, Kai Leung Yung

**Affiliations:** 1 Information Science and Technology Institute, Information Engineering University, Zhengzhou 450001, China; shn4166@163.com (H.S.); wangry@163.com (R.W.); 2 ATR Key Laboratory of National Defense Technology, Shenzhen University, Shenzhen 518060, China; 3 Department of Industrial and Systems Engineering, the Hong Kong Polytechnic University, Hong Kong SAR 999077, China; wh.ip@polyu.edu.hk (W.H.I.); kl.yung@polyu.edu.hk (K.L.Y.)

**Keywords:** trusted cloud, intel sgx, memory protection, eTPM, user-specific

## Abstract

Today cloud computing is widely used in various industries. While benefiting from the services provided by the cloud, users are also faced with some security issues, such as information leakage and data tampering. Utilizing trusted computing technology to enhance the security mechanism, defined as trusted cloud, has become a hot research topic in cloud security. Currently, virtual TPM (vTPM) is commonly used in a trusted cloud to protect the integrity of the cloud environment. However, the existing vTPM scheme lacks protections of vTPM itself at a runtime environment. This paper proposed a novel scheme, which designed a new trusted cloud platform security component, ‘enclave TPM (eTPM)’ to protect cloud and employed Intel SGX to enhance the security of eTPM. The eTPM is a software component that emulates TPM functions which build trust and security in cloud and runs in ‘enclave’, an isolation memory zone introduced by SGX. eTPM can ensure its security at runtime, and protect the integrity of Virtual Machines (VM) according to user-specific policies. Finally, a prototype for the eTPM scheme was implemented, and experiment manifested its effectiveness, security, and availability.

## 1. Introduction

Cloud computing makes precious computing resources become easily available and at a low cost, and its prominent characteristic is ‘supplement on-demand’ [1]. The rapid development and widespread application of cloud computing have brought a high convenience to people, but it also poses new challenges to data security. While the multi-tenancy technology of cloud makes resources shared, the tenant data faces serious threats which make users focus on data security issues. Potential risks, such as malicious programs and untrusted threats underlying cloud environments, have caused many cloud data leakage incidents [2,3,4] in recent years. So the cloud security protections [5,6,7,8] are indispensable for the information society.

A trusted computing [9] platform can be used to protect the integrity of the cloud [10]. Building a virtualization Trusted Platform Module (vTPM) [11] by using Virtualization Technology is an effective method to deal with security threats posed by multi-tenancy of the cloud [12,13,14]. Multiple vTPM instances are created in the cloud platform, at the same time bottom-up trust chains and certificate chains are built to protect the integrity of cloud platforms and clients.

**Figure 1:**
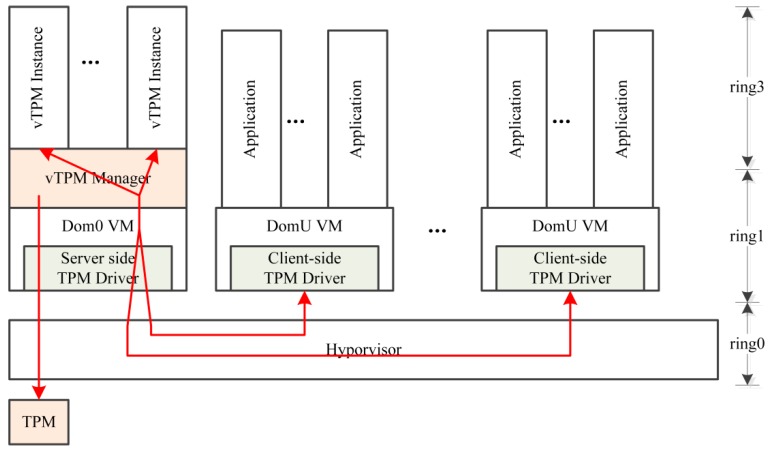
Virtual Trusted Platform Module (vTPM) architecture. VM: virtual machine.

The vTPM architecture in the XEN [15,16], an open source virtual machine monitor developed by Cambridge University, is shown in Figure 1. The vTPM manager runs in the privileged domain Dom0, is responsible for creating and managing vTPM instances at its upper layer, and establishes user-to-vTPM interactions through the front-end and back-end drive mechanism. This scheme can ensure the secure storage of non-volatile random access memory (NVRAM), and can also effectively bind the vTPMs to the virtual machines (VMs).

Although the virtualized TPM provides trusted computing services to enhance the security of the cloud, in reality there are still attacks against vTPM that threaten the security of the cloud: (1) Attackers can abuse vTPM to get sensitive data from users because of the binding relationship between the vTPM and the VM relies on the plaintext configuration file [11], which can easily be tampered with; (2) vTPM runs on Dom0, but the isolation mechanism based on Dom0 and hypervisor does not provide a secure execution environment [17,18]. The sensitive data during the running of the vTPM instance is not guaranteed, and attackers can steal vTPM runtime memory data through memory leak attack; (3) The integrity protection provided by the vTPM is relatively simple and cannot meet the various security requirements of cloud users. Users may need security policies according to their own requirements. It is not only necessary to verify the integrity of the application in the virtual machine, but also to limit the bootstrap sequence of the applications (for example, the anti-virus software needs to be started first). At this stage, vTPM obviously cannot meet this demand.

Intel^®^ Software Guard Extensions (Intel^®^ SGX) [19,20,21,22,23,24] is an extension of the Intel Instruction Set Architecture (ISA) to enhance software security. SGX technology is of great significance to the security of cloud computing. It mainly provides instructions for creating a Trusted Execution Environment (TEE)—Enclave. This method encapsulates the secure operation of legitimate software in an enclave, and the CPU protects it against malicious software attacks, even privileged software cannot access the enclave. Enclave’s security boundary contains only the CPU and itself. In theory, SGX can be widely used in many ways [25,26]; a significant usage is to prevent the compromised OS from attacking the software.

In order to solve the problems in security vulnerabilities and functional limitations of the current vTPM scheme, this paper designed a new trusted cloud platform security component ‘eTPM’ which emulates TPM and implements user-specific functions of trusted cloud based on Intel SGX technology. eTPM is a security component running in the enclave with a user-specific feature, strong cryptographic algorithms, and simulates TPM’s trusted functions. The user-specific feature of eTPM makes users can add user-specific security functions to eTPMs according to their needs. Binding relationships between the eTPM instances and the users are verified by the CPU. Intel SGX technology provides runtime protection that can prevent malicious users or Virtual Machine Manager (VMM) from attacking the eTPM instances running in enclaves.

The paper is structured as follows: Section 2 summarized the related work. Section 3 introduced our SGX-based eTPM scheme for Trusted Cloud Platform. Section 4 presented eTPM security technologies. Section 5 designed a prototype system experiment and verified its effectiveness, security and availability. Finally, Section 6 outlined the conclusions and directions for future work.

## 2. Related Work

In 1971, the concept of trusted computing was firstly proposed at the first International Conference on Fault-Tolerant Computing. In 1985, the U.S. Department of Defense developed the first ‘Trusted Computer System Evaluation Criteria’. In 2003, Trusted Computing Group (TCG) released the new specification V1.2 of the TPM [27].

Virtualization technology provides resource abstraction, isolation, and supplement on-demand for cloud platforms. In the cloud architecture, VMs are the basic units of shared resource distribution, and are also closely related to cloud users. The combination of trusted computing and virtualization provides a security mechanism for the cloud platform and VMs. Berger [11] proposed a TPM virtualization method that can map a physical TPM into multiple vTPMs. Stumpf F [28] proposed a solution for constructing a trusted virtual platform, implements the binding of vTPM to physical TPM, and the construction of vTPM certificate chain. Yuan [29] proposed a vTPM security improvement scheme based on the KVM architecture, which encrypts and protects the vTPM non-volatile storage file by using the simulator ‘Qemu’. This scheme ensures the storage security of NVRAM and solves the binding problem between the vTPMs and the VMs, however, the configuration files that establish the binding relationships are not protected, and the data of the vTPM instances are not protected at runtime. Mainstream virtualization platforms including Xen and KVM all have excellent support for vTPM, while the current vTPM is mainly based on the TPM1.2 standard, which causes the vTPM digest algorithm (SHA1) have a low-security strength and difficulty of managing a wide variety of critical certificates. He et al. [30] proposed a user-specific TPM (abbr. as μTPM) scheme which makes virtualized TPM instances more suitable for cloud environments, by providing users with policy configuration capabilities, but also failed to provide μTPM runtime protection. Fortino G. et al. [31] designed a trust model and an algorithm to form agent groups by using trust measures in the Cloud of Things. Messina F et al. [32] proposed a reputation-based model capable to support the composition of complex Cloud services by taking into account both costs and measures of Quality of Service (QoS). A Iyengar et al. [33] presented a cloud-based system for health care applications that offers enhanced security and privacy over existing systems. Baumann et al. [34] defined the concept of shielded execution and present Haven, the first system to implement shielded execution of unmodified binaries for a commodity OS based on SGX. Arnautov et al. [35] described SCONE, a secure container mechanism for Docker that uses the SGX trusted execution support of Intel CPUs to protect container processes from out-side attacks. Fetzer et al. [36] presented the approach pursued in the context of SERECA1 project to secure micro service based applications based on SGX technology. Brenner et al. [37] investigated the integration of trusted execution based on SGX into microservice applications and presented *Vert.x Vault*, which provides the benefits of micro service architectures together with trusted execution to support privacy and data confidentiality for sensitive applications in the cloud at scale.

## 3. Enclave TPM Scheme

Intel SGX technology can guarantee the trusted execution of Enclave applications based on hardware CPU protection, and it is explicitly designed for multi-core systems that can be multi-threaded and can execute multiple enclaves in parallel, which meets cloud computing requirements. Therefore, with regard to the security vulnerabilities in the current vTPM scheme, this paper introduces SGX technology, building multiple user-specific software TPM—eTPMs and loading them into the enclaves for protection. Firstly, simplify functions of TPM, retain the basic capabilities like measurement, encryption and decryption, sealed storage, and build a more secure and manageable structure. Secondly, add user-specific security configuration referring to μTPM. Finally, make eTPM runs in the TEE to ensure its runtime trusted.

### 3.1. Architecture

#### 3.1.1. Enclave TPM Introduction

eTPM is the core component of the scheme based on SGX. It establishes bottom-up trust chains to protect the integrity of the cloud platform and provides high-strength key algorithms for VMs. The user-specific feature of eTPM makes users can add user-specific security functions to eTPMs according to their needs. And eTPM can protect VM’s sensitive data at runtime. For details on the functions and security mechanisms of eTPM, see Section 4.

#### 3.1.2. Enclave TPM Scheme Architecture

**Figure 2:**
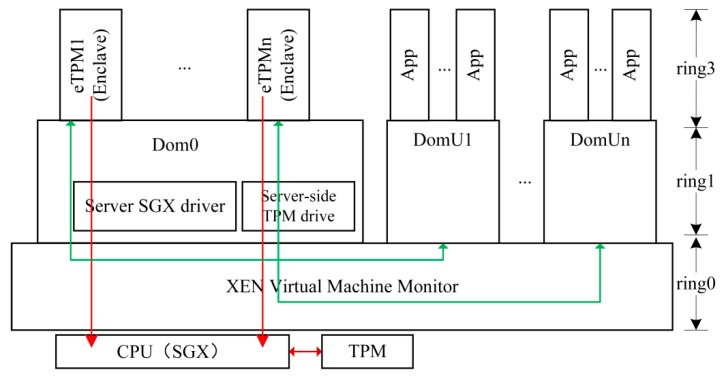
Enclave TPM (eTPM) architecture.

eTPMs are built based on the XEN platform and protected by Intel SGX. It consists of the basic functions such as measurement, key services, secure storage, and user-specific feature. Figure 2 shows system architecture, as we can see, the eTPM is loaded on the privileged domain ‘Dom0’ through the SGX driver of the server, the eTPM and the client VM are bound by the identity seal key. The physical TPM [38] measures the underlying platform. The integrity of the eTPM instances themselves are verified by the CPU, and the eTPM instances measure and protect the user VMs. Intel SGX technology ensures trusted execution of the eTPMs, and users interact with the eTPMs through event channels.

The eTPM system architecture is divided into four levels: (1) The physical hardware layer is equipped with a hardware TPM and a CPU supporting Intel SGX technology. TPM provides physical trust root for the underlying platform. And SGX provides memory protection for the upper platform trusted root ‘eTPM’; (2) In VMM layer (ring0), VMM provides environment isolation and secure communication channels for upper-layer virtual machines through shared memory and event channels, and it is responsible for creating, initializing, deleting and managing virtual machines; (3) In the kernel layer (ring1), the SGX driver module can create enclaves, add or remove enclave pages, and destroy enclaves; (4) In the application layer (ring3), the eTPM runs in the enclave, an isolation area, and is dynamically protected by SGX.

### 3.2. Trust Chains

**Figure 3:**
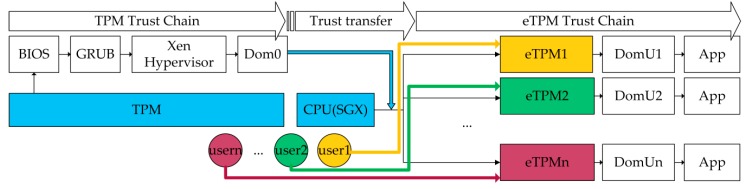
eTPM trust chains.

As shown in Figure 3, this paper proposed a two-level trust chain. The TPM is responsible for establishing the trust chain of the underlying platform; the eTPM creates the upper-level trust chain. The two trust chains are connected in the eTPM which is responsible for verifying the measurement results of the underlying platform. At the same time, a trust relationship between user and eTPM instance is established through the authentication mechanism. The description of the trust chains are as follows:

(1) TPM measures the components of the underlying platform and constructs the underlying platform trust chain step by step according to the order of TPM→CRTM/BIOS→GRUB→XEN Hypervisor→Dom0.
(2) The eTPM runs above Dom0 and constructs the upper-level trust chain in the order of eTPM→DomU→App. For the eTPM, the Trusted Computing Base (TCB) only contains the CPU and the eTPM itself, and the CPU will measure and verify the integrity of the eTPM during its initialization. The eTPM protected by the SGX may be considered as security at runtime. The system architecture consists of two roots of trust, TPM and eTPM, which constitutes to the platform’s underlying trust chain and platform’s upper-level trust chain respectively. In order to transfer trust from underlying platform to up-level platform, the eTPM internal verification module verifies the measurement result of the TPM trust chain and feeds back the verification result only to the VM. This method also avoids the problem of leakage of underlying platform privacy caused by users’ acquisition of underlying platform measurement data.
(3) Each user can configure and extend eTPM instance and control the VMs according to their security requirements. And eTPM authentication mechanism which establishes the trust between users and eTPMs is described in detail in Section 4.4.

### 3.3. User-Specific Feature

The vTPM provides users with integrity protection and key services. However, for cloud users, vTPM mainly has the following two problems: (1) The integrity of the vTPM itself should be measured by the TPM. But resources of the physical TPM are limited, and the bootstrap sequence of a large number of vTPMs in the cloud environment is not static, which makes the TPM unable to meet the integrity measurement of the vTPMs. vTPM, as the root of trust of user VM client, will lead to the interruption of the trusted chain if its integrity is not guaranteed; and (2) In the cloud environment, the security requirements of each user are different (e.g., system component bootstrap sequence and security policy), but vTPM can only provide basic integrity protection that cannot meet the personalized needs of cloud users.

For personalized needs of cloud users, eTPM is designed as user-specific. The eTPM is configurable, as shown in Table 1. The user can configure the eTPM’s personalized protection functions according to requirements, and can also write other security policy codes to extend the eTPM.

Measurement and verification: The eTPM can measure the integrity of a component and verify the result, prevent tampered components from starting.

Static protection: The VM OS image is encrypted when the VM is shut down, and it is decrypted when VM is launched. And sensitive data in the VM can also be processed by using encryption, decryption, or seal functions.

Dynamic protection: Seal store or instant destruct the temporary data generated during eTPM trusted execution.

Components bootstrap: Strictly control the bootstrap of components, services, and applications in the VM. Specify the bootstrap sequence, and prohibit the bootstrap of undefined components.

Personalized requirements: In addition to configuring the above security measures in eTPM, users can also expand eTPM according to their own needs and SGX programming standards. For instance, user can check if the configuration of the access control policies has been tampered with and the access control policies are forced to start. User can also limit the bootstrap sequence of the applications (for example, the anti-virus software needs to be started first).

## 4. Enclave TPM

The eTPM is a core component of this architecture. Multiple cryptography related modules of the eTPM are built by using the SGX trusted cryptography library ‘SGX_tservice’ provided by Intel. The eTPM is divided into two parts, a trusted part handling sensitive data and an untrusted part interacting with the cloud user. SGX-based hardware isolation mechanism protects the trusted part of the eTPM at its running time. In this section, we describe the eTPM in detail.

### 4.1. Enclave TPM Functions

**Figure 4:**
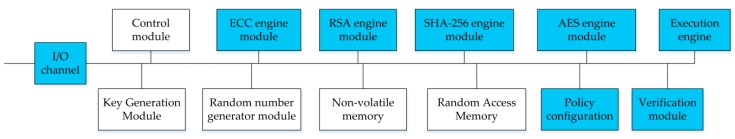
eTPM functional structure.

The TPM is a chip embedded in the main board, with independent executable units, and is a core component of trusted computing. In practical applications, the TPM can be used in real hardware or can be simulated by software, such as TPM_emulater, vTPM. The eTPM designed in this paper is functionally similar to vTPM that provides functions such as key generation, integrity measurement, and sealed storage. The eTPM functions are shown in Figure 4, it simulates TPM in software and uses more secure cryptographic algorithms.

There are some important functional modules specially designed to support the scheme: (1) The verification module is responsible for verifying and reporting the integrity measurement results. An important use of this module is to transfer trust from underlying platform to up-level platform and avoid the problem of leakage of underlying platform privacy, as described in Section 3.2; (2) The policy configuration module is used to configure user-specific security policies that users can enhance the protection of VMs according to their own needs. During eTPM development, users can configure or write specific policies. And eTPM can additionally protect VMs based on specific policies; (3) The execution engine mainly runs the program code and completes operations such as eTPM initialization and measurement; and (4) The I/O channel is mainly responsible for the exchange of commands and messages between the eTPM and the VM. It also establishes the interaction between the eTPM trusted part and untrusted part. Regarding cryptographic modules, the more secure cryptographic algorithms like SHA256, RSA3072, ECC256, and AES are used to provide high-intensity cryptographic services for VMs. And other modules offer functions similar to TPM to protect cloud platform.

### 4.2. Enclave TPM Memory Isolation Mechanism

**Figure 5:**
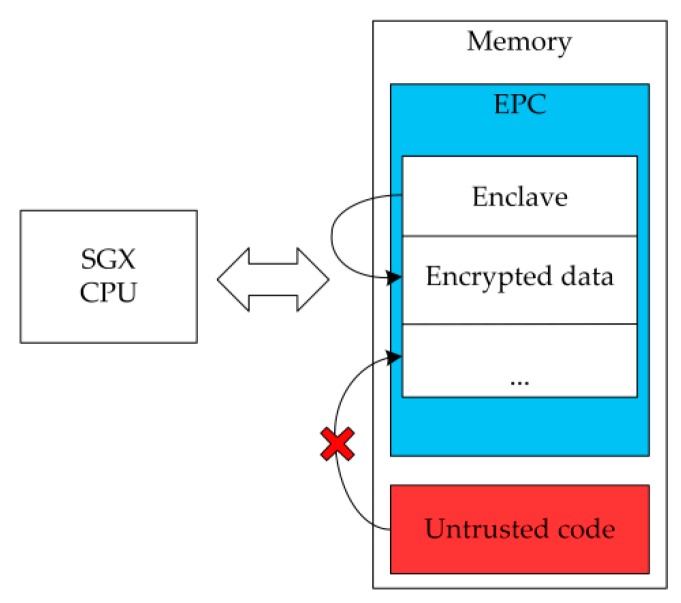
Intel SGX memory isolation mechanism.

eTPM is a software component based on Intel SGX technology and is protected by the CPU directly. It can be securely executed in the Enclave without being attacked by a malicious OS or hypervisor (VMM). When the processor accesses data in the Enclave, it will switch to a new CPU mode called enclave mode automatically. As shown in Figure 5, the enclave mode enforces additional hardware checks for each memory access. Enclave Page Cache (EPC) is an encryption memory area which can prevent data placed in it from being attacked (e.g., memory sniffing). The memory contents in the EPC are encrypted by the memory encryption engine (MEE) and will be decrypted only when entering the CPU package.

Using the memory isolation mechanism provided by SGX, an enclave is firstly created in Dom0 through the SGX driver before creating an eTPM, and then the eTPM library will be loaded into this isolation area. The eTPM instance code and data are stored in the EPC, so that the sensitive information in the eTPM instance can be isolated from the ordinary memory space. SGX physical memory isolation and memory access control mechanisms can ensure that other software, including privileged software, cannot access this isolation area. Intel SGX is designed specifically for multi-core systems that enclaves can be multi-threaded, which enables parallel execution of multiple eTPMs, it meets cloud computing requirements of running multiple VMs at the same time. The data of vTPM instance stored in the memory is in the form of plaintext which is easily being attacked. While the physical memory isolation mechanism of the eTPM can effectively protect sensitive data from memory leak attacks, thereby the confidentiality of memory data at eTPM runtime can be protected.

### 4.3. Enclave TPM Interaction Interface Designs

In the interaction interface of SGX enclave and external applications, there two types of function calls are defined: (1) ECALL: ‘Enclave Call’ a call made from outside application to within the enclave; and (2) OCALL: ‘Out Call’ a call made from within the enclave to the outside application. Because the enclave has no system I/O function and does not provide system function interfaces, developers need to use the OCALL functions to jump to the system space if they want to use the system functions.

#### 4.3.1. Enclave TPM Internal Interaction Interfaces

**Figure 6:**
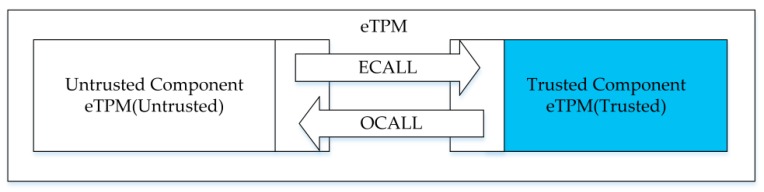
eTPM internal interaction interfaces.

As shown in Figure 6, the eTPM consists of two parts, an internal trusted library ‘eTPM(Trusted)’ and an external application ‘eTPM(Untrusted)’. The trusted library runs inside the enclave and is composed of sensitive code, sensitive data, key-related contents, etc. ECALL is called when an external application requires a trusted execution of the eTPM. And when an I/O and logic processing function is required in the enclave, OCALL is called to enter the system space to interact with the VM and transfer parameters.

#### 4.3.2. Enclave TPM Main Function Call

The important interaction interfaces design between the internal trusted library and the external application of the eTPM are shown in Table 2. These interfaces are mainly related to key-related operations, calculation result outputs, and instruction transmissions.

### 4.4. Enclave TPM’s Integrity

As the root of trust upper-level trust chain of the cloud platform, the integrity of eTPM itself is very important. eTPM signature is a self-signed certificate of the eTPM user, namely SIGSTRUCT. SIGSTRUCT includes eTPM measurement, VM user public key, security version number (SVN), product ID, and so on. And these security attributes of SIGSTRUCT ensure the eTPM itself can be trusted in many aspects. The eTPM measurement attribute is related to the integrity of the eTPM itself.

When the eTPM is placed in the EPC, the CPU measures the eTPM to generate a 256-bit hash, and stores it in the MRENCLAVE register. The CPU compares the value of MRENCLAVE with the eTPM measurement (reference value) in SIGSTRUCT, if the result is matched, we can conclude that the eTPM is correctly loaded into the EPC and can be trusted. The CPU allows eTPM initialization only after the integrity verification satisfies expectations. Measurement and verification of the eTPM by CPU can ensure the integrity of the eTPM itself.

**Figure 7:**
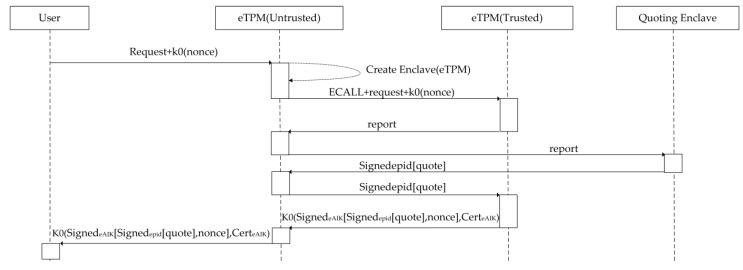
eTPM remote attestation.

In addition to integrity verification of the eTPM provided by the CPU, we also designed a remote attestation mechanism for the eTPM, as shown in Figure 7. This platform is specifically established the Quoting Enclave (QE) for remote attestation. QE is responsible for generating quotes and signing quotes, and Intel provides Intel^®^ Enhanced Privacy ID (Intel^®^ EPID) for signing enclave quotes. Remote attestation process is as follows:

(1) User request: The user sends the request and random number ‘nonce’ to eTPM(Untrusted).
(2) Initialize the eTPM: The eTPM(Untrusted) creates and initializes the eTPM(Trusted). During the initialization process, the CPU verifies the integrity and identity of the eTPM itself.
(3) Attestation: The session key ‘k0’ is agreed in advance between the user and the eTPM. And ‘eAIK’ is eTPM’s signing key. eTPM(Untrusted) executes an ECALL instruction to enter eTPM(Trusted) and then forwards request and a random number encrypted by k0 to eTPM(Trusted). Then the remote attestation report issued by eTPM(Trusted), are forwarded to the platform signing component QE by the eTPM(Untrusted). QE authenticates the report, converts the report to ‘quote’, and signs the ‘quote’ with EPID, then sends it back to eTPM(Trusted) through eTPM(Untrusted). Then eTPM(Trusted) signs the quote signed by EPID and random number ‘nonce’ with eAIK, and encrypts them and eAIK certificate together with k0, and then sends them to the user through eTPM(Untrusted). The user can verify the eTPM after receiving the ‘quote’, eAIK certificate and ‘nonce’.

### 4.5. Enclave TPM Binding Relationships

#### 4.5.1. Identity Binding

SIGSTRUCT, which is cryptographically signed by the eTPM user, includes some important contents such as eTPM measurement, VM user public key, SVN, and product ID. This signature ensures that the eTPM itself is trusted and establishes the binding relationship between the eTPM and the VM user from multiple aspects. The main attributes of SIGSTRUCT are described below.

Enclave measurement: A 256-bit hash that identifies the eTPM’s code, initial data, and their expected sequence and location. 

The user’s public key: After the successful initialization of the enclave, the CPU will record the hash of the eTPM user’s public key in the MRSIGNER register. The contents of MRSIGNER will be used as the eTPM user’s identity. 

SVN: The eTPM user assigns a SVN to the eTPM. The SVN reflects the level of the eTPM security attributes. After the user adds security policies to the eTPM and improves its security level, the SVN should be increased monotonously. 

Product ID: The user assigns a product ID to his eTPM to distinguish the user’s other enclave programs.

**Figure 8:**
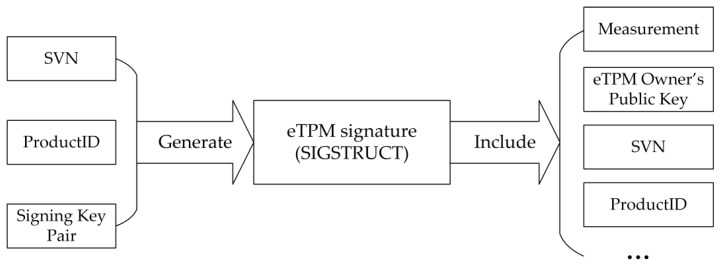
eTPM signature.

As shown in Figure 8, during the development phase, the eTPM user provides the SVN, product ID, and signature key pair for generating the SIGSTRUCT. The CPU uses the public key of the signing key pair to derive the eTPM user identity and then uses the private key to sign the eTPM.

The eTPM performs integrity and identity authentication during its bootstrap: (1) The CPU firstly measures and verifies the integrity of the eTPM placed in the EPC as described in Section 4.4. Then eTPM initialization is allowed; (2) After the eTPM is initialized, the CPU records the hash of the eTPM user’s public key in the MRSIGNER register as the eTPM user’s identity; (3) And the CPU records the SVN assigned by eTPM user which indicates the eTPM security level; and (4) Similarly, the CPU records the Product ID.

The VM is in an encrypted state before it is launched and can only be decrypted by the corresponding eTPM. The VM is encrypted by the eTPM when shutting down. Bootstrap process of VM as follows: (1) The user logs in the cloud platform and requests to launch the eTPM; (2) After the eTPM completes the identity and integrity check, it is allowed to be initialized and launched; and (3) The corresponding VM image need be decrypted, measured and verified by eTPM before eTPM launches the VM. 

This architecture establishes identity binding relationships between eTPM, users, and guest VMs through eTPM user key pairs, SIGSTRUCT, and VM bootstrap control mechanism. And the identity binding relationship is guaranteed by CPU: The eTPM can be initialized only when the integrity and identity are verified by CPU.

#### 4.5.2. Data Binding

Enclave has two sealing policies: (1) Seal to the current enclave. The seal key is bound to the current version of the enclave measurement (MENCLAVE). Only enclaves with the same MRENCLAVE measurement value can unseal the sealed data; and (2) Seal to the enclave author. When the Enclave is initialized, the CPU stores the author’s identity in the MRSIGNER register, and then binds the MRSIGNER value and product ID to the seal key. Only when the enclave matches the MRSIGNER register value and has the same product ID, can unseal the sealed data.

The first kind of sealing policy makes the sealed data valid only for the current undisturbed enclave; The second sealing policy binds the sealed data with the identity of the enclave author (corresponds to eTPM user), so that the user can update the eTPM and the sealed data is still valid. At the same time, the sealed key between each VM cannot be mixed.

eTPM use the method ‘sealing to the enclave author’ has the following two benefits: (1) eTPM instance and user sensitive data are tightly bound because sealed data can only be unsealed in the eTPM with the correct identity, that helps prevent attackers (malicious users) from stealing users’ confidential information by misusing the eTPM instances; and (2) Sensitive data are bound to the user’s identity rather than eTPM measurement value. This is more suitable for our user-specific eTPM, for the reason that the sealed data can still be unsealed after user updates eTPM. 

### 4.6. Enclave TPM Key Management

#### 4.6.1. Key Derivation

**Figure 9:**
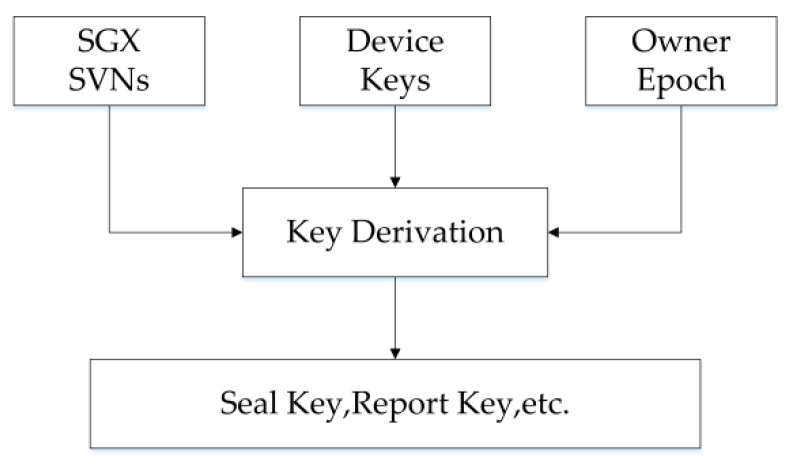
Key derivation.

The eTPM platform designed in this paper requires three attributes for key derivation: SVNs, Device Keys, and Owner Epoch. The device key is a 128-bit number bound to the processor, represents the environment of the hardware platform; Owner Epoch is the key that Intel assigns to the owner of the platform, giving the owner ability to add key entropy values. As shown in Figure 9, the derivation of the eTPM key is jointly determined by the VM user, the device, and the platform service provider. And this mechanism enables the platform owner (cloud service provider) to change all the keys in the system through owner epoch. In particular, when the platform is migrated or transferred, the purpose of denying others access to sealed data can be achieved by changing/restoring the owner epoch.

#### 4.6.2. Cryptographic Algorithm

The eTPM supports cryptographic algorithms such as SHA256, AES (rijndael128), ECC256, and RSA3072. Compared with vTPM’s major algorithms like SHA-1, eTPM’s key algorithm has higher security. In addition, the user-specific eTPM allows users to add more cryptographic algorithms.

### 4.7. Enclave TPM Trusted Execution

**Figure 10:**
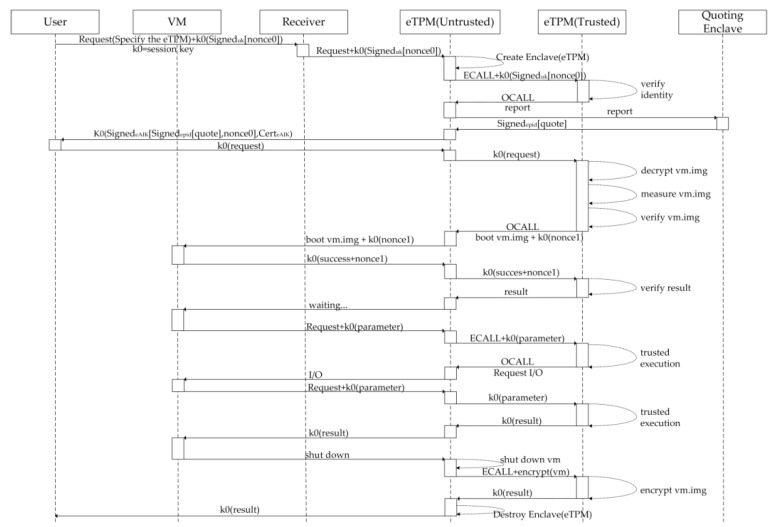
eTPM work flow.

The workflow of eTPM is shown in Figure 10. The eTPM user requests to load the corresponding eTPM instance, then the eTPM user and eTPM instance authenticate each other after the eTPM instance is initialized; If the authentication is passed, the eTPM decrypts, measures, and launches the VM image; After the VM is launched, a session between the VM and the eTPM is established, then VM can use the eTPM to perform key-related or user-specific trusted executions; When the user needs to shut down the VM, a request is sent to the eTPM, and then the eTPM encrypts and stores the user VM image. 

The process can be divided into four phases: (1) Bootstrap of eTPM; (2) Bootstrap of VM; (3) eTPM trusted execution; and (4) VM and eTPM shutdown phase. The formal description of the process is as follows:

(1) Bootstrap of eTPM:

$$\begin{array}{l} Message1\;\;\;User\to Receiver:Request,\{[nonce0]{}_{uk^{-1}}\}{}_{K0}\\ Message2\;\;\;Receiver\to eTPM(Untrusted):Request,\{{\left[nonce0\right]}_{uk^{-1}}\}{}_{K0}\\ Message3\;\;\;eTPM(Untrusted)\to eTPM(Trusted):\{[nonce0]{}_{uk^{-1}}\}{}_{K0}\\ Message4\;\;\;eTPM(Trusted)\to eTPM(Untrusted):report\\ Message5\;\;\;eTPM(Untrusted)\to QE:report\\ Message6\;\;\;QE\to eTPM(Untrusted):{\left[quote\right]}_{EPID^{-1}}\\ Message7\;\;\;eTPM(Untrusted)\to User:\{[{\left[quote\right]}_{EPID^{-1}},nonce0{]}_{eAIK^{-1}},Cert_{eAIK}\}{}_{K0} \end{array}$$

The user requests the cloud server’s ‘Receiver module’ to launch the specified eTPM, and this module forwards the remote authentication request, the random number signed by user’s identity key ‘uk’ and encrypted by session key ‘k0’ to the eTPM. After the eTPM(Untrusted) is launched by the Receiver module, the eTPM(Trusted) starts to be initialized, and the CPU will verify the integrity and identity of the eTPM itself during initialization. Then The eTPM(Untrusted) sends an ECALL instruction to enter eTPM(Trusted) and forwards the encrypted and signed nonce to eTPM(Trusted). After the identity of the user verification by eTPM(Trusted) is passed, perform the eTPM remote attestation at Section 4.4.

(2) Bootstrap of VM:

$$\begin{array}{l} Message1\;\;\;User\to eTPM(Untrusted):\{Request\}{}_{K0}\\ Message2\;\;\;eTPM(Untrusted)\to eTPM(Trusted):{\left\{Request\right\}}_{K0}\\ Message3\;\;\;eTPM(Trusted)\to eTPM(Untrusted):{\left\{nonce1\right\}}_{K0}\\ Message4\;\;\;eTPM(Untrusted)\to VM:{\left\{nonce1\right\}}_{K0}\\ Message5\;\;\;VM\to eTPM(Untrusted):{\left\{success,nonce1\right\}}_{K0}\\ Message6\;\;\;eTPM(Untrusted)\to eTPM(Trusted):{\left\{success,nonce1\right\}}_{K0}\\ Message7\;\;\;eTPM(Trusted)\to eTPM(Untrusted):result \end{array}$$

eTPM starts to launch the VM after receiving the request. The eTPM(Trusted) decrypts, measures and verifies VM image. After the verification is passed, the OCALL instruction is issued to notify the eTPM(Untrusted) to launch the user VM image, and forward ‘k0(nonce1)’ to the VM through eTPM(Untrusted). After the VM starts up, the message ‘k0(success+nonnce1)’ is sent to eTPM(Untrusted), then the message is forwarded to eTPM(Trusted) as a parameter of OCALL. After the eTPM(Trusted) verifies the message of the launched VM, it completes the VM bootstrap and returns the bootstrap result to the eTPM(Untrusted). Then eTPM(Untrusted) turns to the listening state.

(3) eTPM trusted execution:

$$\begin{array}{l} Message1\;\;\;VM\to eTPM(Untrusted):Request,{\left\{parameter\right\}}_{K0}\\ Message2\;\;\;eTPM(Untrusted)\to eTPM(Trusted):{\left\{parameter\right\}}_{K0}\\ Message3\;\;\;eTPM(Trusted)\to eTPM(Untrusted):{\left\{result\right\}}_{K0}\\ Message4\;\;\;eTPM(Unrusted)\to VM:{\left\{result\right\}}_{K0} \end{array}$$

The eTPM(Untrusted) waits for user instructions after the VM is launched. When eTPM is needed, the user sends the request and parameter ‘k0(parameter)’ to eTPM(Untrusted). The eTPM(Untrusted) calls correlation function of the eTPM(Trusted) through ECALL for trusted executions. Part of the trusted executions is shown in Table 3.

(4) VM and eTPM shutdown phase:

$$\begin{array}{l} Message1\;\;\;VM\to eTPM(Untrusted):shutdown\\ Message2\;\;\;eTPM(Untrusted)\to eTPM(Trusted):encrypt\\ Message3\;\;\;eTPM(Trusted)\to eTPM(Untrusted):{\left\{result\right\}}_{K0}\\ Message4\;\;\;eTPM(Untrusted)\to User:{\left\{result\right\}}_{K0} \end{array}$$

When the user is ready to shut down the VM, it sends a shutdown request to the eTPM(Untrusted). The eTPM(Untrusted) shuts down the VM and sends an ECALL to encrypt VM image. Then the eTPM(Trusted) encrypts the VM image and returns the ECALL result ‘k0(result)’. And the eTPM(Untrusted) executes the command to destroy eTPM(Trusted) and returns ‘k0(result)’ to the user. The user can confirm the shutdown process by verifying the result.

## 5. Experiments and Analysis

This paper described the development of a prototype system for the eTPM scheme based on Intel SGX technology. The system platform configuration is as follows: Intel(R) Core(TM) i7 CPU; TPM v1.2; Intel Linux SGX driver and SDK v2.11; XEN para-virtualized platform v4.4; Dom0 kernel version: Linux 4.5.2; DomU kernel version: Linux 3.9.1. At present, Intel’s latest SGX patch package supports the virtualization of SGX on KVM and XEN platforms. It also supports running the enclave in VMs. However, this article considered the following reasons to choose to run the enclave on the privileged domain: (1) If virtualized SGX technology is used in VMs, the VM image cannot be measured before the system starts; and (2) At present, SGX technology only supports up to 128 MB memory. If this technology is used in each VM, it may cause the problem of overload.

Experiments in this chapter are to verify the effectiveness, security, and availability of the eTPM system. (1) The effectiveness of eTPM was verified through simulation experiments on eTPM workflow; (2) The security of eTPM was analysed through formal proof of remote attestation protocol, memory protection experiment and sealed data protection experiment; (3) The availability of eTPM was analysed by comparing performance with other trusted cloud schemes. And the experimental results and conclusions are based on the assumption that SGX is secure.

### 5.1. Enclave TPM Effectiveness

**Figure 11:**
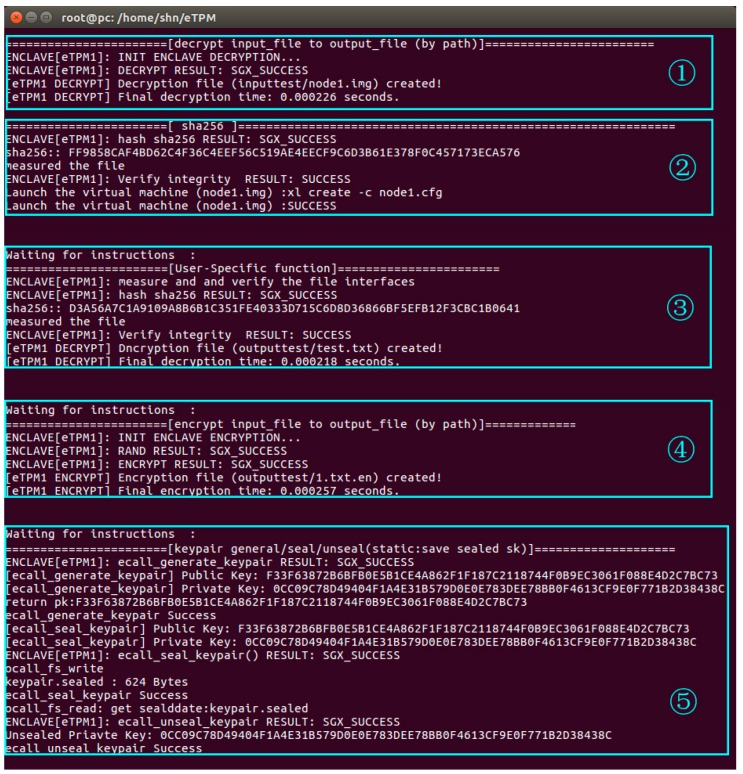
eTPM1 work flow experiment.

The experiment in this section verified the effectiveness of the eTPM through the simulation of the workflow shown in Figure 10. The experiment based on the assumption that the user and the eTPM completed the mutual authentication already. The experiment in this section completed the trusted bootstrap of the user’s VM by the eTPM, applied a personalized security policy, and requested the eTPM to process sensitive data, as shown in Figure 11.

The experimental process is described as follows:

(1) The eTPM firstly decrypts the image of the VM ‘node1’.
(2) The eTPM1 measures the image of node1 and that the measured values match the reference values. Then node1 is launched and a random number ‘nonce1’ encrypted by the session key ‘K0’ is sent to node1.
(3) After node1 is launched, a session with the eTPM1 is established and then a message that indicates VM launched successfully is sent to eTPM1 by node1. When eTPM1 receives this feedback message, it changes into the state of ‘waiting for instructions’.

The node1 user has defined the personalized security policy for eTPM1 before: The file of node1 can be decrypted only if the network configuration file of node1 has not been tampered. When eTPM1 receives the request for decrypting the file, it firstly checks the network configuration file of node1. After the verification is passed, eTPM1 decrypts the file.

(4) eTPM1 encrypts the file according to the request of encrypting a file.
(5) eTPM1 generates, seals, and unseals a key pair according to the request.

This experiment implements some of the processes in Figure 10 that completes the trusted bootstrap of the user’s VM by eTPM, applies a personalized security policy, and requests the eTPM to process sensitive data. Experimental results demonstrate the effectiveness of eTPM from functional implementation.

### 5.2. Enclave TPM Security

This section proves the security of the protocol through SVO logic formalization, and verifies memory security and storage data security through memory access experiments and sealed data access experiments.

SGX does have vulnerabilities in practice. The existing problems are mainly concentrated on: (1) putting malicious code into the enclave to cause data leakage in the memory protection area; (2) SGX technology suffers from side channel attacks. But for the following reasons we assume that SGX is securely in the system: (1) The initialization of the eTPM is verified by the CPU. Therefore, we believe that eTPM is trustworthy and there is no such thing as malicious code entering the enclave; (2) The cloud infrastructure is the responsibility of the service provider. We believe that the physical facilities of the data center are highly secure and can avoid the behaviour of physical attacks.

#### 5.2.1. Protocol Security

The eTPM workflow includes remote attestation and trusted executions related to keys and sensitive data. Taking remote attestation as an example, a short formal proof and analysis of remote attestation protocol based on SVO logic [39,40], a kind of BAN-like logic, was given. The core protocols in remote proof are as follows:

$$\begin{array}{l} Message1\;\;\;User\to eTPM:\{{\left\{nonce\right\}}_{uk^{-1}}\}{}_{K0}\\ Message2\;\;\;eTPM\to QE:report\\ Message3\;\;\;QE\to eTPM:{\left[quote\right]}_{EPID^{-1}}\\ Message4\;\;\;eTPM\to User:\{[{\left[quote\right]}_{EPID^{-1}},nonce]{}_{eAIK^{-1}},Cert_{eAIK}\}{}_{K0} \end{array}$$

The protocol should achieve the following objectives: User can verify the identity of the message sender and can verify the freshness of the message to prevent replay attacks during the communication. The SVO logic is used to prove and analyse the eTPM remote attestation mechanism. And the result shows that the method can achieve the desired objective and prevent replay attack. 

The symbols and meanings commonly used in SVO logic are shown in Table 4.

The symbols used in this paper are explained as follows:

$K^{-1}$: The decryption key corresponding to the key K.

${\left\{X^{P}\right\}}_{K}$: Message X encrypted into a ciphertext by key *K*. P is the sender (usually omitted).

${\left[X\right]}_{K^{-1}}$: Message X signed by $K^{-1}$, i.e., ${\left[X\right]}_{K^{-1}}=X,{\left\{H(X)\right\}}_{K^{-1}}$. H() is the hash function.

PK(P,K): K is the public key of the principal P, and $K^{-1}$ is the corresponding private key.

$PK_{\sigma }(P,K)$: K is the public encryption key of principal P.

SV(X,K,Y): The key K can be used to verify that X is the signature of Y.

$P\overset{k}{↔}Q$: K is a good shared key between P and Q.

SVO logic divides the language into message language $M_{\Gamma }$ and formula language $F_{\Gamma }$ on set Γ. Γ is the set of atomic terms, consisting of a set of constant symbols that do not intersect each other.

**Definition** **1.** $M_{\Gamma }$ *is the smallest language satisfying:*

*(1)*  X*is a message, If*X∈Γ.
*(2)*  $F(X_{1},\cdots ,X_{K})$ *is a message, if* $X_{1},\cdots ,X_{k}$ *are messages.* F *is any n-dimensional function.*
*(3)*  φ *is a message if* φ *is a formula.*

**Definition** **2.** $F_{\Gamma }$ *is the smallest language satisfying:*

*(1)*  φ *is a formula, if* φ *is a primitive proposition.*
*(2)*  ¬φ *and* φ∧ψ *are formulae(Includes other propositions connected by* ¬ *and* ∧ *), if* φ *and* ψ *are formulae.*
*(3)*  P|≡φ *and* P|⇒φ *are formulae, if* P *is a principal and* φ *is a formula.*
*(4)*  P∍X *,* P⊲X *,* P|∼X *,* P|≈X *and* #(X) *are formulae, if* P *is a principal and* X *is a message.*
*(5)*  $P\overset{k}{↔}Q$ *,* PK(P,K) *and* P∍K *are formulae, if* P *is a principal and* K *is a key.*

SVO logic has two basic inference rules:

Modus Ponens (MP): From φ and φ⊃ψ infer ψ;

Necessitation (Nec): From ├φ infer ├P|≡φ. (The meaning of ├ is explained in Definition 3).

**Definition** **3.** *If* Ω *is a set of initialization assumptions for a protocol, and* Γ *is a set of formulae, then a proof of Ω├Γ in SVO logic means that there is a formulae sequence $F_{1},F_{2},\cdots ,F_{n}$ of limited length such that Γ is a subset of $\left\{F_{1},F_{2},\cdots ,F_{n}\right\}$, and for ∀i∈{1,2,…,n}, $F_{i}$ satisfies one of the following three conditions:*

*(1)*  $F_{i}$ *is an instantiation of an axiom;*
*(2)*  $F_{i}$ *is an assumption;*
*(3)*  $F_{i}$ *can be derived from some of the preceding formulae by using MP or Nec rules.*

If φ├Γ has a proof in the SVO logic, then the conclusion φ├Γ is established. If Ω=ϕ, we can simply write ϕ├Γ as ├Γ. According to the rationality of the SVO logic, there is: if the proposition in ├Γ is all true, then the proposition in Γ is all true.

The axioms and conclusion used are listed below:

(1) Believing:
  A0 (P|≡φ∧P|≡ψ)≡(P|≡(φ∧ψ))
  A1 P|≡φ∧P|≡(φ⊃ψ)⊃P|≡ψ
(2) Receiving:
  A7 $P⊲(X_{1},\cdots ,X_{n})⊃P⊲X_{i}$
  A8 $(P⊲{\left\{X\right\}}_{K}\wedge P∍K^{-1})⊃P⊲X$
  A9 $P⊲{\left[X\right]}_{K}⊃P⊲X$
(3) Seeing:
  A10 P⊲X⊃P∍X
(4) Freshness:
  A18 $\#(X_{i})⊃\#(F(X_{1},\cdots ,X_{n}))$

**Conclusion** **1.** Using the A1 axiom and MP rules, the following common conclusion can be obtained:

A1 + MP (P|≡φ∧P|≡(φ⊃ψ)) ⊃P|≡ψ. 

We assume that the user can verify the eTPM signature (signature of eAIK), the eAIK’s certificate ‘CerteAIK’ and the platform signature (signature of the EPID). eTPM and QE use Secure Channel for messaging within the platform. And there is a good session key K0 between the user’s eTPM. Assumption set as follows, where principal *U* stands for the user, principal E stands for eTPM, and principal *P* stands for platform:

P1 $U|\equiv PK_{\sigma }(E,eAIK)$
P2 $U|\equiv PK_{\sigma }(P,EPID)$
P3 U|≡U∍eAIK
P4 U|≡U∍K0
P5 U|≡U∍EPID
P6 $\begin{array}{l} \left(U∍eAIK\wedge (U|\equiv PK_{\sigma }(E,eAIK))\wedge U∍{\left[{\left[quote\right]}_{EPID^{-1}},nonce1\right]}_{eAIK^{-1}}\right)\\ ⊃SV([{\left[quote\right]}_{EPID^{-1}},nonce]{}_{eAIK^{-1}},eAIK,({\left[quote\right]}_{EPID^{-1}},nonce)) \end{array}$
P7 $\left(U∍EPID\wedge (U|\equiv PK_{\sigma }(P,EPID))\wedge U∍{\left[quote\right]}_{EPID^{-1}})⊃SV({\left[quote\right]}_{EPID^{-1}},EPID,quote\right)$
P8 U|≡#(nonce)
P9 $U⊲{\left\{[{\left[quote\right]}_{EPID^{-1}},nonce{]}_{eAIK^{-1}},Cert_{eAIK}\right\}}_{K0}$
P10 $D|\equiv U⊲{\left\{{\left[{\left[quote\right]}_{EPID^{-1}},nonce1\right]}_{eAIK^{-1}},Cert_{eAIK}\right\}}_{K0}$

Formal proof and analysis as follows:

Available from assumptions P10, P4 and Axiom A0:

(1) $$U|\equiv U⊲{\left\{[{\left[quote\right]}_{EPID^{-1}},nonce{]}_{eAIK^{-1}},Cert_{eAIK}\right\}}_{K0}\wedge U∍K0.\;$$

Instantiate the axiom A8 and available from Nec rule:

(2) $$\begin{array}{l} U|\equiv (U⊲\{[{\left[quote\right]}_{EPID^{-1}},nonce{]}_{eAIK^{-1}},Cert_{eAIK}\}{}_{K0}\wedge U∍K0).\\ ⊃U⊲({\left[{\left[quote\right]}_{EPID^{-1}},nonce\right]}_{eAIK^{-1}},Cert_{eAIK}). \end{array}$$

Available from Equations (1) and (2) and conclusion A1 + MP:

(3) $$U|\equiv U⊲({\left[{\left[quote\right]}_{EPID^{-1}},nonce\right]}_{eAIK^{-1}},Cert_{eAIK}).\;$$

Instantiate the axiom A7 and available from Nec rule:

(4) $$U|\equiv U⊲({\left[{\left[quote\right]}_{EPID^{-1}},nonce\right]}_{eAIK^{-1}},Cert_{eAIK})⊃U⊲{\left[{\left[quote\right]}_{EPID^{-1}},nonce\right]}_{eAIK^{-1}}.\;$$

Available from Equations (3) and (4) and conclusion A1 + MP:

(5) $$U|\equiv U⊲{\left[{\left[quote\right]}_{EPID^{-1}},nonce\right]}_{eAIK^{-1}}.\;$$

Instantiate the axiom A9, A10 and available from Nec rule:

(6) $$U|\equiv U⊲{\left[{\left[quote\right]}_{EPID^{-1}},nonce\right]}_{eAIK^{-1}}⊃U⊲[{\left[quote\right]}_{EPID^{-1}},nonce].\;$$

(7) $$U|\equiv U⊲{\left[{\left[quote\right]}_{EPID^{-1}},nonce\right]}_{eAIK^{-1}}⊃U∍{\left[{\left[quote\right]}_{EPID^{-1}},nonce\right]}_{eAIK^{-1}}.\;$$

Available from Equations (5) and (7) and conclusion A1 + MP:

(8) $$U|\equiv U∍{\left[{\left[quote\right]}_{EPID^{-1}},nonce\right]}_{eAIK^{-1}}.\;$$

Available from assumptions P3, P1, Equation (8) and axiom A0:

(9) $$U|\equiv (U∍eAIK\wedge (U|\equiv PK_{\sigma }(E,eAIK))\wedge U∍{\left[{\left[quote\right]}_{EPID^{-1}},nonce\right]}_{eAIK^{-1}}).\;$$

Instantiate the assumption P9 and available from Nec rule:

(10) $$\begin{array}{l} U|\equiv (U∍eAIK\wedge (U|\equiv PK_{\sigma }(E,eAIK))\wedge U∍{\left[{\left[quote\right]}_{EPID^{-1}},nonce\right]}_{eAIK^{-1}}).\\ ⊃SV({\left[{\left[quote\right]}_{EPID^{-1}},nonce\right]}_{eAIK^{-1}},eAIK,({\left[quote\right]}_{EPID^{-1}},nonce)). \end{array}$$

Available from Equations (9) and (10) and conclusion A1 + MP:

(11) $$U|\equiv SV({\left[{\left[quote\right]}_{EPID^{-1}},nonce\right]}_{eAIK^{-1}},eAIK,({\left[quote\right]}_{EPID^{-1}},nonce)).\;$$

Instantiate the axiom A18 and available from Nec rule:

(12) $$U|\equiv \#(nonce)⊃\#({\left[{\left[quote\right]}_{EPID^{-1}},nonce\right]}_{eAIK^{-1}}).\;$$

Available from assumption P8, Equation (12) and conclusion A1 + MP:

(13) $$U|\equiv \#({\left[{\left[quote\right]}_{EPID^{-1}},nonce\right]}_{eAIK^{-1}}).\;$$

The following result is easily obtained in the same way:

(14) $$U|\equiv SV({\left[quote\right]}_{EPID^{-1}},EPID,quote).\;$$

For the principal U, the following results are obtained according to the Equations (11), (13) and (14). The results show that: User believes that the remote attestation report is signed by the corresponding eTPM and platform, and believes the report is fresh:

$$U|\equiv SV({\left[{\left[quote\right]}_{EPID^{-1}},nonce\right]}_{eAIK^{-1}},eAIK,({\left[quote\right]}_{EPID^{-1}},nonce)),\;U|\equiv \#({\left[{\left[quote\right]}_{EPID^{-1}},nonce\right]}_{eAIK^{-1}}),\;U|\equiv SV({\left[quote\right]}_{EPID^{-1}},EPID,quote).\;$$

By using SVO logic to briefly prove the remote attestation protocol, we can conclude that: User can verify identity of the message sender and can verify the freshness of the message to prevent replay attacks during the remote attestation. 

#### 5.2.2. Memory Security

The eTPM stores and processes sensitive data in its isolated area, so that attackers cannot steal data in the eTPM through memory sniffing. In this section and Section 5.2.3, we defined three roles, namely user A: the eTPM1 user, user B: the VMM privileged administrator, and user C: eTPM2 user. The experiments simulate these three roles to access the sensitive data in the current eTPM memory space respectively, and the experimental results prove that the eTPM has capabilities of runtime protection.

**Figure 12:**
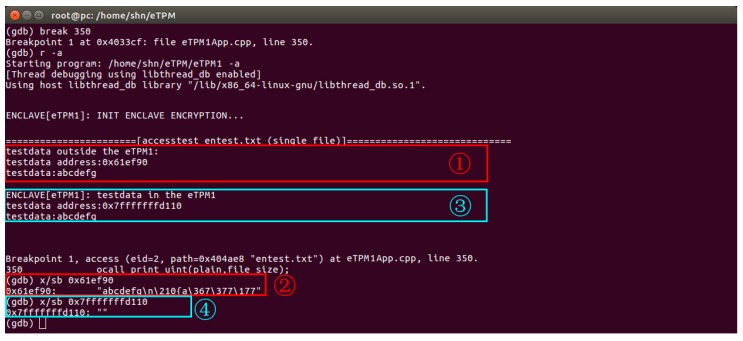
Memory data test.

**Figure 13:**
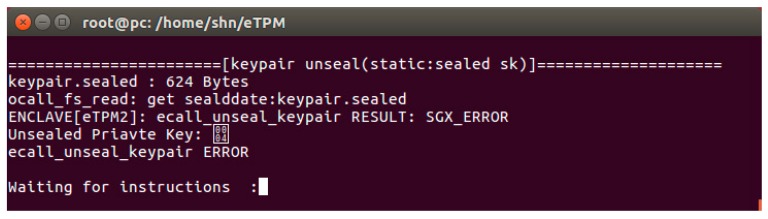
Storage data test.

Figure 12 shows an experiment in which user A and user B attempt to access data of current eTPM1 respectively. As shown in Figure 13, ① A non-sensitive data stored outside the memory isolation area, and user A outputs its content and address. ② User B can read this data by accessing the memory address. ③ The eTPM1 sensitive data are stored in the isolated memory area, user A outputs the address and content of the data through the OCALL interface. ④ User B attempts to access the data in eTPM1 through the address, as a result, the data cannot be read. This experiment proves that the eTPM runtime sensitive data is only accessible to current eTPM users. Even if the VMM privileged administrator cannot access sensitive data, it proved that the eTPM can protect the data at runtime.

#### 5.2.3. Data Security

The eTPM can also encrypt or seal its data, it is impossible for attackers to steal static data at rest. The following experiment shows that an attacker cannot obtain sensitive data from users by abusing eTPM.

Figure 13 is an experiment that user C trying to read the sealed key pair of user A through his eTPM. As shown in Figure 13, the user C uses eTPM2 to unseal the key pair sealed by the eTPM1, and as a result, the operation cannot be performed and the content of the key pair cannot be read efficiently. This experiment proves that there is a binding relationship between the eTPM and the data, the malicious user cannot abuse the eTPMs to steal data which is securely stored by eTPMs of other users.

In this section, the security of the protocol is analysed through formal proof, and the memory security and data security are analysed through experiments. The formal proof demonstrates that user can verify eTPM identity during remote attestation, etc., and the protocols can resist replay attacks during interaction. And experiments above proved that eTPM can resist memory sniffing attacks and can prevent stored files from being attacked. 

### 5.3. Enclave TPM Availability

**Figure 14:**
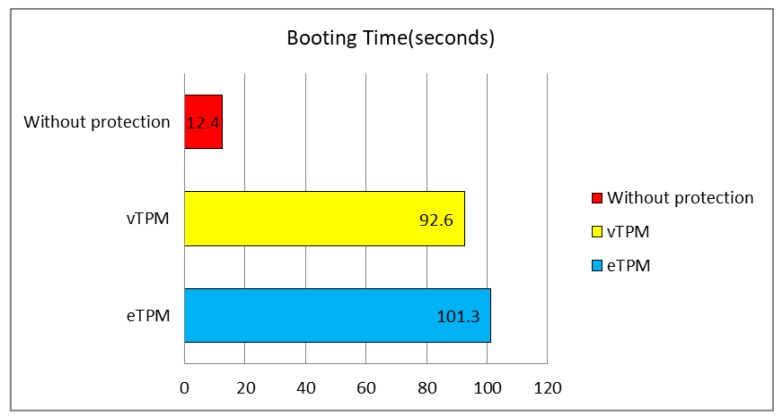
Booting time of VMs.

The experiment in this section verifies the availability of the eTPM by comparing the booting time of the VMs in the eTPM scheme and the vTPM scheme [11] on Xen, and we use booting time of the VMs without protection as the benchmark. We conduct this experiment 20 times and record the average booting time as shown in Figure 14. The result of experimental shows that the booting time of the VMs with protection schemes has increased obviously compared with the benchmark because of the security mechanisms, while increased booting time of the VMs in eTPM scheme is only 8.7 s compared with the vTPM scheme which does not exceed 10%. This experiment proves that the increased performance overhead of the eTPM scheme is within an acceptable range.

Experiments in this chapter are to verify the effectiveness, security, and availability of the eTPM system. The results show that: (1) The effectiveness of eTPM is verified from the perspective of performing functions. And eTPM can effectively implement its workflow. (2) The security of the protocol is analysed through formal proof, and the memory security and data security are analysed through experiments. The formal proof demonstrates that user can verify eTPM identity during remote attestation, etc., and the protocols can resist replay attacks during interaction. And experiments proved that eTPM can resist memory sniffing attacks and can prevent stored files from being attacked. (3) The availability of the eTPM is verified by comparing the booting time of the VMs in the eTPM scheme and the vTPM scheme on xen. And the increased performance overhead of the eTPM scheme is within an acceptable range.

## 6. Conclusions and Future Work

Recently, attacks such as memory sniffing and data tampering have caused a large number of security incidents in the cloud environment. Existing trusted cloud vTPM scheme lacks memory protection and some other vital security protection problems that cannot against such attacks. To resolve the problems, we proposed a novel trusted cloud platform security component ‘eTPM’, and designed a trusted cloud platform scheme based on eTPM. Here key issues in the design and implementation process of eTPM are studied in detail, including its architecture, trust chain, and user-specific capabilities.

Our proposed eTPM scheme focuses on the runtime trusted, key algorithm security, and user-specific feature, besides, the binding relationship between eTPM and VM is protected by hardware. We have elaborated the detail design of the architecture and finally, a prototype system is implemented on the xen; various tests were carried out, and the experiment manifests its effectiveness, security, and availability. 

## Figures and Tables

**Table 1:** eTPM user-specific protection functions.

Functions	Configuration	Description
Measurement and verification	Standard configuration	Measure and verify the integrity of components
Static protection	Standard configuration	Encrypt storage OS image, data
Dynamic protection	Standard configuration	Temporary data security processing
Components bootstrap	User self-configuration	Component bootstrap order limit
Personalized requirement s	User-specific	Such as access control policies

**Table 2:** The main function call.

Function Name	Interaction Interface	Cryptographic Library Interface	Description
ecall_encrypt/ecall_decrypt	ECALL	sgx_rijndael128GCM_encrypt/sgx_rijndael128GCM_decrypt	Performs a 128 bit key size Rijndael AES-GCM encryption/decryption operation
eacll_generater and	ECALL	sgx_read_rand	Generates a random number inside the enclave.
ecall_hash	ECALL	sgx_sha256_msg	Performs a standard SHA256 hash of the input data buffer.
ecall_sealdata/ecall_unsealdata	ECALL	sgx_seal_data/sgx_unseal_data	Use AES-GCM to seal/unseal the input data
ecall_ecckeypair	ECALL	sgx_ecc256_create_key_pair	Generates a private/public key pair on the ECC curve.
ecall_rsasign/ecall_rsaverify	ECALL	sgx_rsa3072_sign/sgx_rsa3072_verify	Calculates/Verify the digital signature for a given data set based on the RSA 3072 private/public key.
ecall_aesctrencyrpt/ecall_aesctrdecrypt	ECALL	sgx_aes_ctr_encrypt/sgx_aes_ctr_decrypt	Performing 128-bit Rijndael AES-CTR encryption/decryption
ecall_report	ECALL	sgx_create_report	Create a cryptographic report that describes the contents of the calling enclave.
ecall_generatekey	ECALL	sgx_get_key	Generates a 128-bit secret key using the input information.
ocall_output	OCALL	/	Output the execution result
ocall_instructions	OCALL	/	Transfer instructions

**Table 3:** Trusted execution.

Trusted Execution	Description
Verify the integrity of the underlying platform	Get the TPM’s measurement values for the underlying platform and verify them in the eTPM, to connect trust chains.
Measure and verify data	Measure and verify user’s data within eTPM
Seal/unseal data	Seal/unseal user’s sensitive data or keys so that the data or keys can only be unsealed in the user’s eTPM.
Encrypt/decrypt data	Use the key algorithm provided by eTPM to encrypt/decrypt user’s data so that data can be securely stored on the hard disk.
User-specific trusted execution	Users can expand eTPM according to their own needs, add specific functions.

**Table 4:** SVO logical symbols.

**Symbol**	|≡	|≈	|∼	⊲	≡	∍	#	⊃	|⇒
meaning	believes	says	said	received	equals	sees/has	fresh()	implies	controls

## References

[1] Mell P., Grance T. (2011). The NIST Definition of Cloud Computing.

[2] Chen Y., Paxson V., Katz R.H. (2010). What’s New about Cloud Computing Security.

[3] Ristenpart T., Tromer E., Shacham H., Savage S. Hey, you, get off of my cloud: Exploring information leakage in third-party compute clouds. Proceedings of the 16th ACM Conference on Computer and Communications Security.

[4] Jiang J., Han G., Shu L., Chan S., Wang K. (2017). A trust model based on cloud theory in underwater acoustic sensor networks. IEEE Trans. Ind. Inform..

[5] Kaufman L.M. (2009). Data security in the world of cloud computing. IEEE Secur. Priv..

[6] Khalil I.M., Khreishah A., Azeem M. (2014). Cloud computing security: A survey. Computers.

[7] Liu Y., Sun Y., Ryoo J., Rizvi S., Vasilakos A.V. (2015). A survey of security and privacy challenges in cloud computing: Solutions and future directions. J. Comput. Sci. Eng..

[8] Coppolino L., D’antonio S., Mazzeo G., Romano L. (2017). Cloud security: Emerging threats and current solutions. Comput. Electr. Eng..

[9] Martin A. (2008). The Ten-Page Introduction to Trusted Computing.

[10] Achemlal M., Gharout S., Gaber C. Trusted platform module as an enabler for security in cloud computing. Proceedings of the IEEE 2011 Conference on Network and Information Systems Security (SAR-SSI).

[11] Berger S., Goldman K.A., Perez R., Sailer R., Doorn L. Vtpm: Virtualizing the Trusted Platform Module. Proceedings of the 15th Conference on Usenix Security Symposium.

[12] Yan Q., Han J., Li Y., Deng R.H., Li T. A software-based root-of-trust primitive on multicore platforms. Proceedings of the 6th ACM Symposium on Information, Computer and Communications Security.

[13] Riad K. Multi-authority trust access control for cloud storage. Proceedings of the IEEE 2016 4th International Conference on Cloud Computing and Intelligence Systems (CCIS).

[14] Garfinkel T., Pfaff B., Chow J., Rosenblum M., Boneh D. Terra: A virtual machine-based platform for trusted computing. Proceedings of the Nineteenth ACM Symposium on Operating Systems Principles.

[15] Takemura C., Crawford L.S. (2009). The Book of Xen.

[16] Xue H., Qing S., Zhang H. (2007). XEN virtual machine technology and its security analysis. Wuhan Univ. J. Nat. Sci..

[17] Garfinkel T., Rosenblum M., Dan B. Flexible OS support and applications for trusted computing. Proceedings of the Conference on Hot Topics in Operating Systems.

[18] Wojtczuk R., Rutkowska J., Tereshkin A. (2008). Xen 0wning Trilogy.

[19] Anati I., Gueron S., Johnson S., Scarlata V. (2013). Innovative technology for CPU based attestation and sealing. Proceedings of the 2nd International Workshop on Hardware and Architectural Support for Security and Privacy.

[20] McKeen F., Alexandrovich I., Berenzon A., Rozas C.V., Shafi H., Shanbhogue V., Savagaonkar U.R. Innovative instructions and software model for isolated execution. Proceedings of the 2nd International Workshop on Hardware and Architectural Support for Security and Privacy.

[21] Hoekstra M., Lal R., Pappachan P., Phegade V., Del Cuvillo J. Using innovative instructions to create trustworthy software solutions. Proceedings of the 2nd International Workshop on Hardware and Architectural Support for Security and Privacy.

[22] Intel Inc. Intel Software Guard Extensions. https://software.Intel.com/en-us/SGX.

[23] Intel Inc. Intel Software Guard Extensions Reference. https://software.Intel.com/sites/default/files/332680-002.pdf.

[24] Schuster F., Costa M., Fournet C., Gkantsidis C., Peinado M., Mainar-Ruiz G., Russinovich M. VC3: Trustworthy data analytics in the cloud using SGX. Proceedings of the 2015 IEEE Symposium on Security and Privacy (SP).

[25] Chang R., Jiang L., Chen W., Xie Y., Lu Z. (2017). A trust enclave-based architecture for ensuring run-time security in embedded terminals. Tsinghua Sci. Technol..

[26] Yan F., Yu Z., Zhang L., Zhao B. (2017). Vtse: A solution of sgx-based vtpm secure enhancement. Adv. Eng. Sci..

[27] (2006). Trusted Computing Group: Trusted Platform Module (tpm) Specifications. Technical Report. https://www.trustedcomputinggroup.org/specs/TPM.

[28] Stumpf F., Benz M., Hermanowski M., Eckert C. (2007). An approach to a trustworthy system architecture using virtualization. Proceedings of the International Conference on Autonomic and Trusted Computing.

[29] Shi Y., Zhao B., Yu Z., Zhang H. (2015). A security-improved scheme for virtual TPM based on KVM. Wuhan Univ. J. Nat. Sci..

[30] Rongyu H., Shaojie W., Lu J. (2013). A User-specific Trusted Virtual Environment for Cloud Computing. Inf. Technol. J..

[31] Fortino G., Fotia L., Messina F., Rosaci D., Sarn G.M. (2018). Forming Groups in the Cloud of Things Using Trust Measures. Proceedings of the International Symposium on Intelligent and Distributed Computing.

[32] Messina F., Pappalardo G., Comi A., Fotia L., Sarn G.M.L., Rosaci D. (2017). Combining reputation and QoS measures to improve cloud service composition. Int. J. Grid Util. Comput..

[33] Iyengar A., Kundu A., Sharma U., Zhang P. A Trusted Healthcare Data Analytics Cloud Platform. Proceedings of the IEEE International Conference on Distributed Computing Systems.

[34] Baumann A., Peinado M., Hunt G. (2014). Shielding Applications from an Untrusted Cloud with Haven. ACM Trans. Comput. Syst..

[35] Arnautov S., Trach B., Gregor F., Knauth T., Martin A., Priebe C., Lind J., Muthukumaran D., O’keeffe D., Stillwell M. SCONE: Secure Linux Containers with Intel SGX. Proceedings of the OSDI.

[36] Fetzer C., Mazzeo G., Oliver J., Romano L., Verburg M. Integrating Reactive Cloud Applications in SERECA. Proceedings of the 12th International Conference on Availability, Reliability and Security.

[37] Brenner S., Hundt T., Mazzeo G., Kapitza R. Secure Cloud Micro Services Using Intel SGX. Proceedings of the IFIP International Conference on Distributed Applications and Interoperable Systems.

[38] Arthur W., Challener D. (2015). A Practical Guide to TPM 2.0: Using the Trusted Platform Module in the New Age of Security.

[39] Syverson P.F., Oorschot P.C.V. On Unifying Some Cryptographic Protocol Logics. Proceedings of the IEEE Symposium on Security and Privacy.

[40] Syverson P. (1996). A Unified Cryptographic Protocol Logic.

